# Mesenchymal stem cells reduce alcoholic hepatitis in mice via suppression of hepatic neutrophil and macrophage infiltration, and of oxidative stress

**DOI:** 10.1371/journal.pone.0228889

**Published:** 2020-02-11

**Authors:** Yue-Meng Wan, Zhi-qiang Li, Chang Liu, Yue-Feng He, Men-Jie Wang, Xi-Nan Wu, Yuan Zhang, Yu-Hua Li

**Affiliations:** 1 Public Health Institute of Kunming Medical University, Kunming, Yunnan province, China; 2 Gastroenterology Department, The 2^nd^ Affiliated Hospital of Kunming Medical University, Kunming, Yunnan Province, China; 3 The Biomedical engineering research center, Kunming Medical University, Kunming Yunnan, China; Medizinische Fakultat der RWTH Aachen, GERMANY

## Abstract

Mesenchymal stem cells (MSCs) are a population of pluripotent cells that have been tested for the treatment of many inflammatory diseases. It remains unclear whether MSCs were effective in treating mice with alcoholic hepatitis (AH) and its underlying mechanism. In the present study, MSCs were isolated from bone marrow of 4–6 week-old C57BL/6N male mice. AH was induced in female mice by chronic-binge ethanol feeding for 10 days. Intraperitoneal (i.p.) transplantation of MSCs or saline were performed in mice on day 10. Blood samples and hepatic tissues were harvested on day 11. Biochemical, liver histological and flow cytometric analyses were performed. Compared to the control mice, the AH mice had significantly increased liver/body weight ratio, serum alanine aminotransferase (ALT) and aspartate aminotransferases (AST), hepatic total cholesterol (TC), triglyceride (TG), malondialdehyde (MDA), hepatic neutrophil and macrophage infiltration (P<0.001), which were markedly reduced by i.p. transplantation of MSCs (P<0.01). Compared to the control mice, the hepatic glutathione (GSH) was prominently lower in the AH mice (P<0.001), which was markedly enhanced after i.p. injection of MSCs (P<0.001). MSCs were effective for the treatment of AH mice, which might be associated with their ability in inhibiting hepatic neutrophil and macrophage infiltration, and alleviating oxidative stress.

## Introduction

Alcoholic liver disease (ALD) is mainly caused by excessive alcohol drinking that induces a variety of hepatic injuries including simple steatosis, steatohepatitis, fibrosis, cirrhosis, and hepatocellular carcinoma (HCC). Over the last 30 years, the incidence of ALD in China has increased significantly, due to its explosive economic growth and increasing social openness that have boosted alcohol consumption [1]. In China, ALD was accountable for 23.4% deaths related to liver cancer in men and 2.2% in women in 2005 [2]. Among all patients admitted to hospital due to liver disease, the percentage of ALD patients climbed up from 1.7% in 2002 to 4.6% in 2013, and the yearly incidence of severe alcoholic hepatitis (AH) also rised up by 2.43 times from 2002 to 2013, according to a survey conducted in China [3].

AH is a necroinflammatory process characterized by poor liver function and impaired hepatocyte regeneration in ALD [4]. At early stage, ALD such as steatosis may disappear after alcohol abstinence in heavy drinkers, whereas AH, the inflammatory lesions in ALD, can persist for several months after alcohol withdrawal [5], which prominently increases the risk of hepatic decompensation and liver cirrhosis [6]. Severe AH is associated with a high short-term mortality that remains a challenge for clinicians due to lack of effective treatment, although corticosteroids have been widely used and TNF-α inhibitors have been tested in treating this severe illness [7,8]. To date, there is still unmet need to explore other effective treatment options for AH or its severe form.

Cytotherpy therapy with mesenchymal stem cells (MSCs) has been attempted in many other model of inflammatory disease, such as inflammatory bowel disease, peritonitis and rheumatoid arthritis [9–11]. MSCs are a heterogeneous population of pluripotent cells that can be obtained and expanded ex vivo from different tissues. They can differentiate along mesenchymal lineages and bear immunomodulatory properties, which make them an ideal source for cell-based regenerative strategies in mesenchymal tissue damage and immune-mediated diseases [12]. Based on these findings, MSCs seem to be a good candidate for the treatment of AH. However, there is very limited data about the use of MSCs for this disorder in the literature.

Therefore, we performed the present study to investigate the effect and its underlying mechanism of MSCs derived from mouse bone marrow (BM) in treating AH in a mouse model.

## Materials and methods

### Mice

30 female 8–10 week-old C57BL/6N mice were purchased from the Experimental Animal Center of Kunming Medical University, and maintained in closed cages (dimensions: 37 cm in length, 26 cm in width, 17 cm in height) padded with wood debris in a ventilated room. The wood debris was changed everyday to keep the padding dry. The mice were randomly allocated to the following three groups of nine animals per cage, and maintained under a 12 h/12h (day/night) light cycle, temperature (21–24°C) and humidity (40–70%) with free access to the ethanol-containing or control liquid diet. All animal care and experimental procedures were performed in accordance with the institutional policies for animal health and well-being, and were approved by the Ethics Committees of Kunming Medical University (No. KMMU 2019067). All animals were monitored every 12 hours to document their activity, hair change and diet.

### Isolation and characterization of MSCs

MSCs were isolated and characterized from the BM of tibias and femurs of male 4–6 week-old C57BL/6N mice according to previous studies [13,14]. MSCs at passage 2–3 were used in the following experiments.

### AH modeling, grouping and treatment

AH was established according to the National Institute on Alcohol Abuse and Alcoholism (NIAAA) protocol [15,16]. The diets were purchased from TROPHIC Animal Feed High-Tech Co. Ltd (No. TP4030C and TP4030D; Hai’an, Jiangsu, China), and were prepared fresh everyday. On day 10, mice in the MSCs group received intraperitoneal (i.p.) injection of BM-MSCs (5×10^6^ cells/mouse in 0.5ml saline), whereas the mice in the control and AH groups only received i.p. 0.5ml/mouse sterile saline. The gavage was performed in the morning, and mice were anesthetized by inhalation of diethyl ether 9 hours following the last gavage. Blood samples were collected after removing eyeballs. Then mice were sacrificed by cervical dislocation, and liver tissues were harvested and stored at -80°Cfor further assays.

### Biochemical analysis

Serum concentrations of alanine aminotransferase (ALT; No.C009-2), aspartate aminotransferases (AST; No.C0010-2), hepatic total cholesterol (TC; No.A111-1) and triglyceride (TG; No.A110-1), hepatic malondialdehyde (MDA; No.A003-1) and glutathione (GSH; No.A006-2) were all measured using spectrophotometric assay kits (NanJing JianCheng Bioengineering Institute, Jiangsu, China) according to the manufacturer’s instructions.

### Flow cytometric assay

Flow cytometry was performed on a Partec GmbH CyFlow Space system using CyView software (Partec, Germany). Briefly, for immunophenotypical characterization of MSCs derived from mouse BM, the cells at passage 2–3 were harvested after digestion with 0.25% trypsin (Gibco, USA; No.1894145) and resuspended in phosphate-buffered saline (PBS; Sigma, USA; No.P5493). Subsequently, the cells were incubated with the following antibodies: rat anti-mouse antibodies against CD90-phycoerythrin (PE) (Abcam, USA; No.ab24904), CD29-fluorescein isothiocyanate (FITC) (Abcam, USA; No.ab21845), CD45-TITC (Invitrogen, USA; No.11-0451-82), CD105-TITC (Invitrogen, USA; No.MA5-17945) as previously described [17]. For analysis of hepatic neutrophil and macrophage infiltration, fresh liver tissues were cut into small blocks (about 1mm^3^) and grounded using grinding slides in a petri dish with PBS. After centrifugation at 4°C,1000rpm for 5 minutes, the pellets were digested using 0.25% trypsin (3-5ml) at 37°C for 20 minutes. Following stoppage of digestion by serum-containing culture medium, the cell suspensions were passed through a 70 μm cell strainer and centrifuged at 4°C, 1000rpm for 5 minutes. The pellets were resuspended in serum-free culture medium and centrifuged (4°C, 1000rpm ×5min) again. The cell pellets were then resuspended in 100μl PBS and incubated at dark, room temperature with 5μl rabbit anti-mouse anti-Ly-6G-FITC (Invitrogen, USA; No. 11-5931-82) for 30minutes to detect neutrophils or with 5μl rat anti-mouse anti-CD11b-allophycocyanin (APC; Abcam, UK; No.ab25482) and 5μl anti-CD206-PE (Invitrogen, USA; No.12-2061-80) to detect macrophages, as previously described [18].

### Myeloperoxidase (MPO) activity measurement

Liver tissue was homogenized, and hepatic MPO activity was measured using a MPO assay kit (NanJing JianCheng Bioengineering Institute, Jiangsu, China; No.A044) in a U-3010 UV-Vis Spectrophotometer (HITACHI, Japan) according to the manufacturer’s instruction. MPO activity was expressed in units per gram of wet tissues, and one unit indicates the enzyme activity needed to degrade 1μM H_2_O_2_/min/ml at 24°C.

### Liver histology

Liver specimens were fixed in 10% paraformaldehyde, embedded in paraffin and cut into 5μm sections. Specimens were dewaxed, hydrated, and stained with standard hematoxylin and eosin (H&E) for routine histology. H&E sections were scored by an experienced pathologist (blind to the group allocation) according to a previously validated scoring system (S1 Table) [19,20]. Briefly, the histology scores for hepatic steatosis, hepatocyte ballooning and necroinflammatory activity were calculated and averaged from 9 random 200× fields per liver section from the same mouse. For assessment of lipid accumulation, 10 μm sections were cut from frozen samples and stained with Oil Red O **(**Beijing solarbio science & technology co.,ltd., China; No.G1260).

### Statistical analysis

Data are presented as means ± standard deviation (SD) and were analyzed using GraphPad PRISM software, version 8.02 (GraphPad Software Inc, La Jolla, CA, United States). A Kruskal-Wallis one-way ANOVA followed by Tukey’s post hoc test (for three or more group comparisons) was used for statistical analyses. A value of P<0.05 was considered statistically significant.

## Results

### Morphology, phenotype and differentiation profiles of MSCs

**Fig 1:**
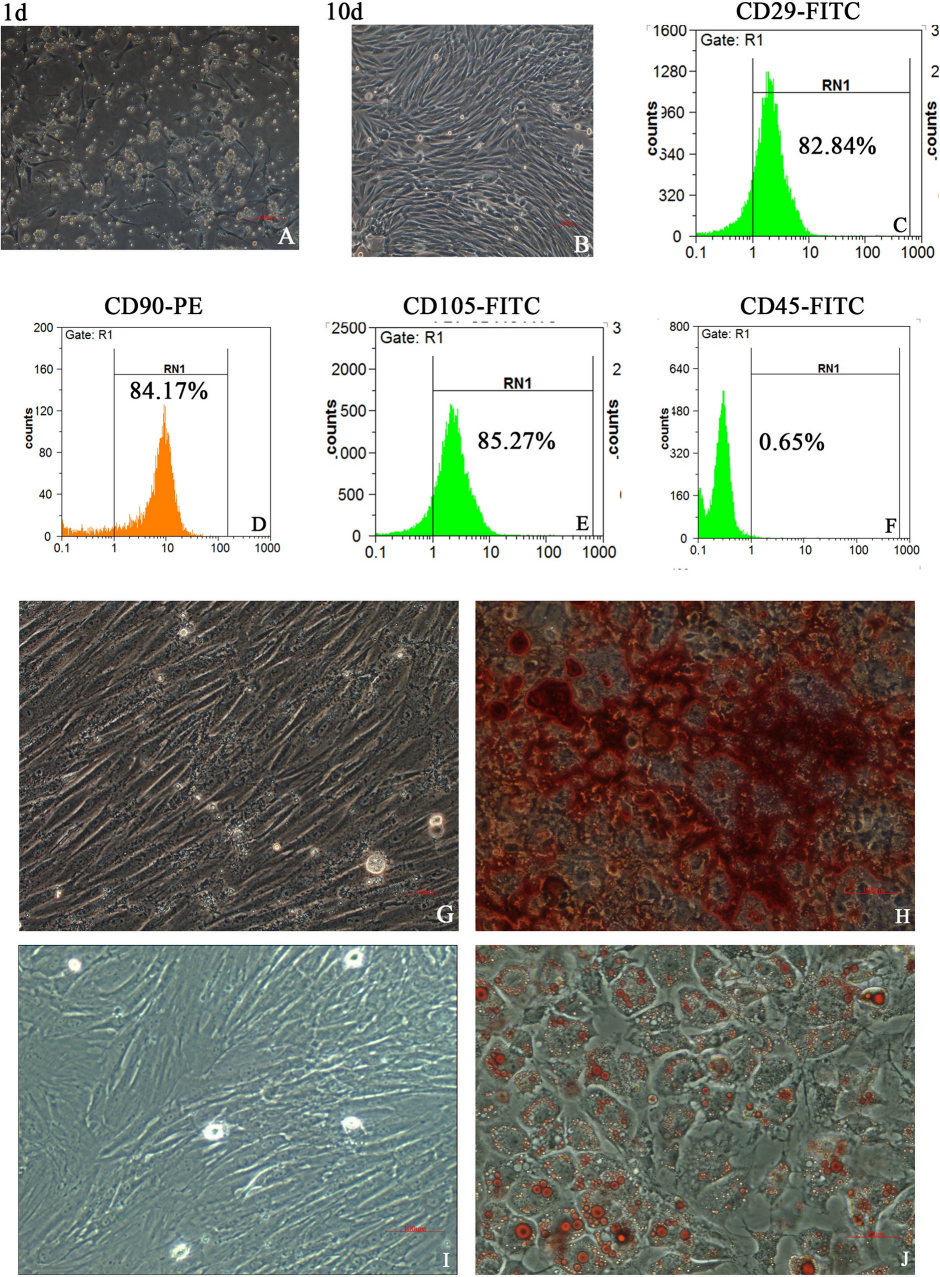
A-J The primary plastic adherent cells isolated from mouse bone marrows showed a spindle-shape morphology after culture for 1 day before changing culture medium (A) and 10 days after changing culture medium (B); immunophenotypical characterization by flow cytometry at passage 2–3 showed positivity for CD29 (C), CD90 (D) and CD105 (E), but negativity for CD45 (F); osteogenic (G,H) and adipogenic (I,J) differentiation assays revealed that the isolated cells had undergone osteogenic differentiation (bone nodules formation illustrated by red staining) before (G) and after (H) alizarin red staining, and adipogenic differentiation (accumulation of lipid droplets illustrated by red staining) before (I) and after (J) oil red O staining. Bars = 100μm. FITC, fluorescein isothiocyanate; PE, phycoerythrin.

The isolated adherent cells displayed a spindle-shape fibroblast-like morphology (Fig 1A and 1B) after culture for 6–10 days. They expressed high-level CD29 (82.84%; Fig 1C), CD90 (84.17%; Fig 1D) and CD105 (85.27%; Fig 1E), but low-level hematopoietic marker CD45 (0.65%) (Fig 1F). They had the ability to differentiate into osteoblasts (Fig 1G and 1H) and adipocytes (Fig 1I and 1J) after culture in osteogenic and adipogenic mediums. These features were consistent with the typical MSCs, but not hematopoietic cells [14,21].

### General condition of mice

**Fig 2:**
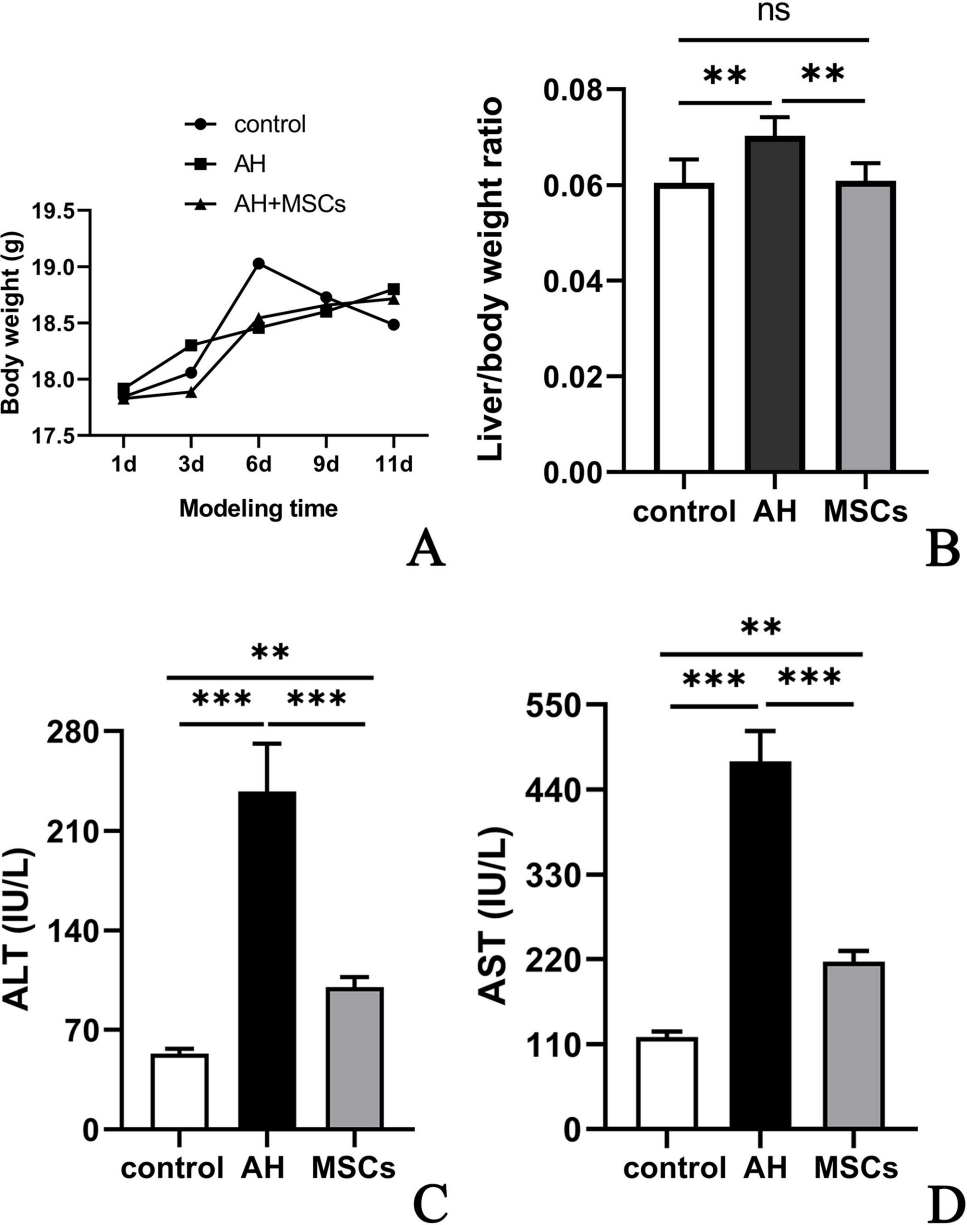
A-D MSCs attenuated ethanol induced liver injury: body weight change (A), liver/body weight ratio (B), serum ALT(C) and AST (D) levels. Five to ten mice per group were used. *P<0.05, **P < 0.01, ***P < 0.001. ALT, alanine aminotransferase; AST, aspartate aminotransferase; MSCs, mesenchymal stem cells.

During the modeling period, mice in all groups were similar in general activity, hair and body weight (Fig 2A; S2A Table). After alcohol garage, mice were lethargic and tachypneic, but recovered completely the next day.

### MSCs attenuated ethanol-induced liver injury

Compared to the control group, the AH group had significantly elevated liver/body weight ratio (Fig 2B; S2B Table), serum ALT (Fig 2C; S2C Table) and AST (Fig 2D; S2D Table) levels (P<0.001), which were significantly decreased after treatment with i.p. MSCs (P<0.01).

### MSCs reduced liver steatosis and hepatic lipid content

**Fig 3:**
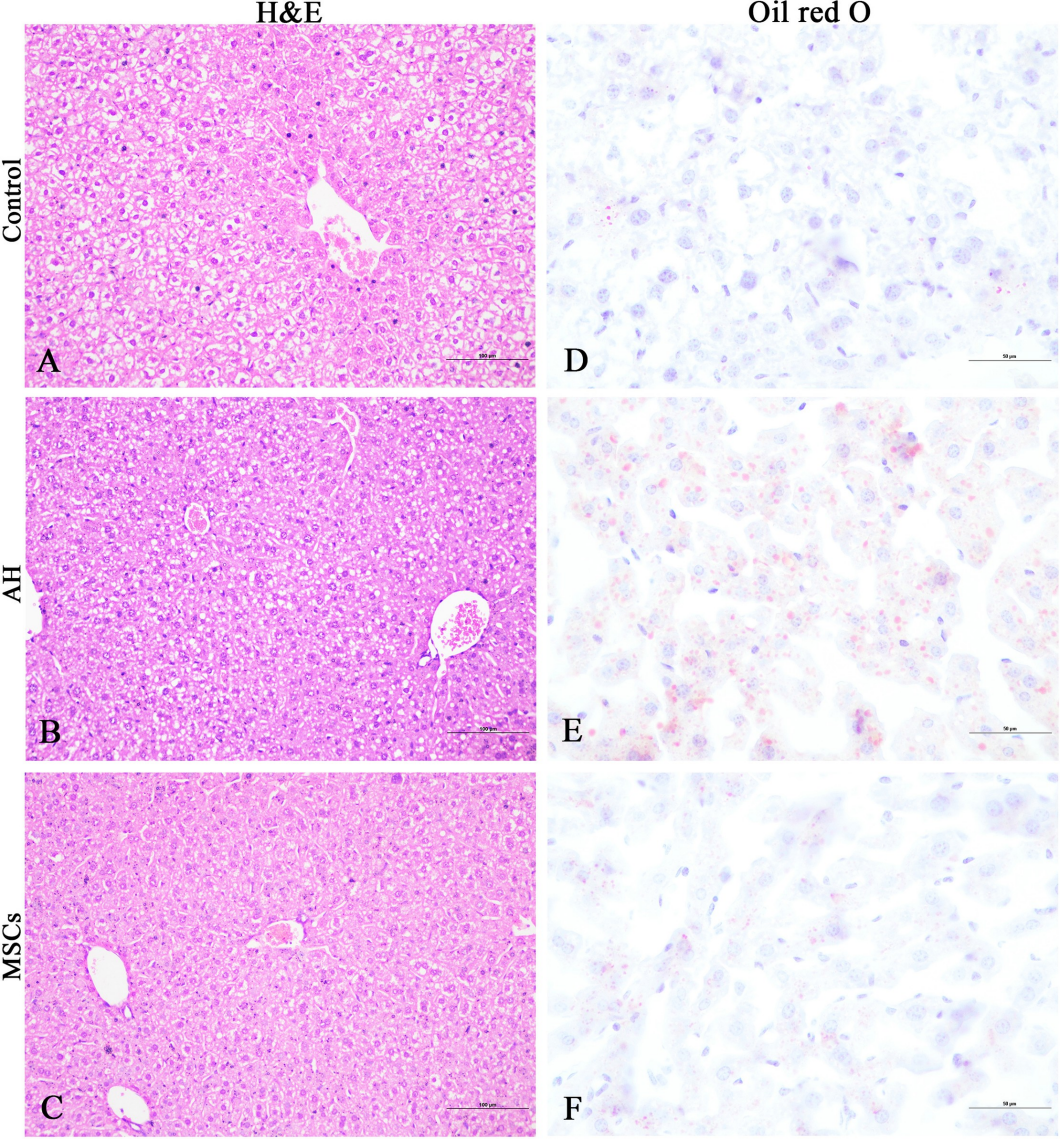
A-F MSCs reduced liver steatosis: hematoxylin & eosin staining for control (A), AH (B), MSCs (C) groups; oil red O staining for control (D), AH (E), MSCs (F) groups. Bars = 100μm (A-C) or 50μm (D-F). AH, alcoholic hepatitis; MSCs, mesenchymal stem cells.

**Fig 4:**
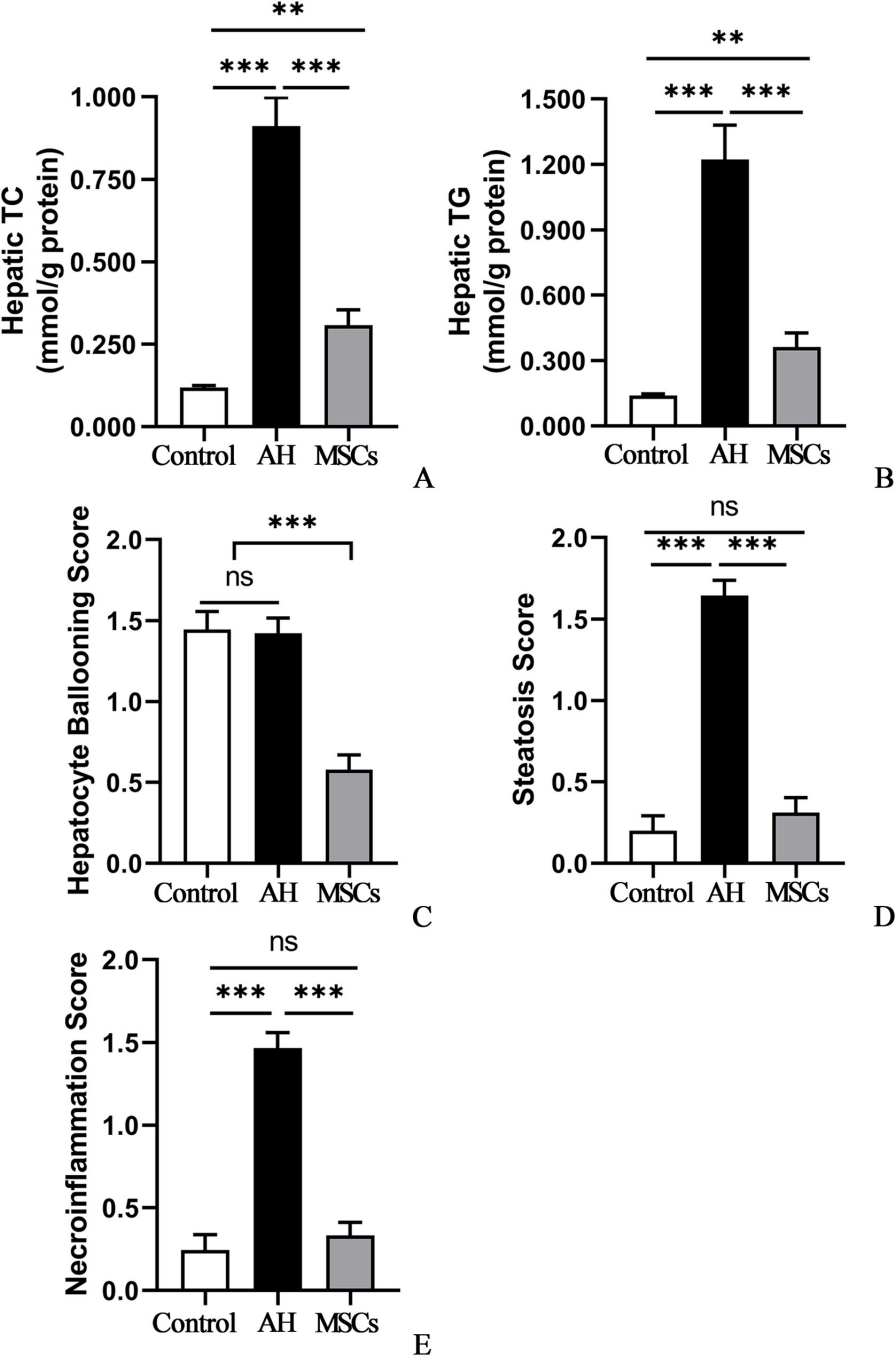
A-E MSCs reduced hepatic lipid accumulation, and improved liver histology: hepatic TC (A) and TG (B) contents; hepatocyte ballooning score (C), steatosis score (D) and necroinflammation score (E). Five mice per group were used. *P < 0.05, **P < 0.01, ***P < 0.001. MSCs, mesenchymal stem cells; TC, total cholesterol; TG, triglyceride.

Compared to the control group, the AH group showed prominent swelling or ballooning hepatocytes, disorderly hepatocyte cord, micro- and macrovesicular steatosis as revealed by H&E (Fig 3A–3C) and oil red O staining (Fig 3D–3F). Hepatic lipid contents measurement revealed that the AH had significantly higher TC (P<0.001; Fig 4A; S3A Table) and TG (P<0.001; Fig 4B; S3B Table) levels than the control group, which were markedly reduced after after i.p. infusion of MSCs (P<0.001; Fig 4A and 4B; S3A and S3B Table). Histological analysis further showed that the AH group had similar hepatocyte ballooning score (P>0.05; Fig 4C; S3C Table) but significantly higher scores for steatosis (P<0.05; Fig 4D; S3D Table) and necroinflammation (P<0.05; Fig 4E; S3E Table) than the control group, which were markedly reduced after i.p. infusion of MSCs (P<0.05; Fig 4C–4E).

### MSCs alleviated hepatic oxidative stress

**Fig 5:**
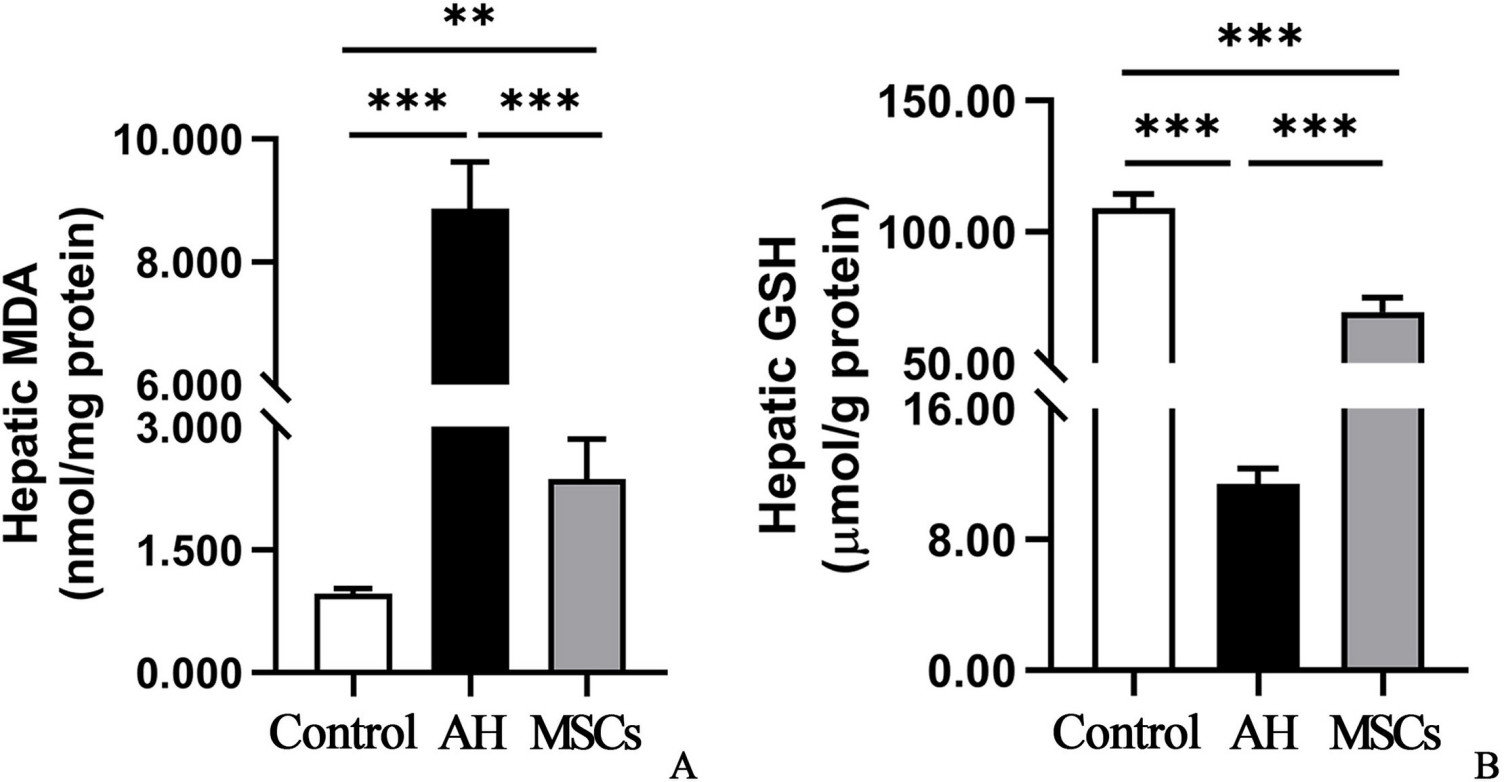
A,B MSCs alleviated hepatic oxidative stress: hepatic MDA (A) and GSH (B) contents. Five mice per group were used. *P < 0.05, **P < 0.01, ***P < 0.001. GSH, glutathione; MDA, malondialdehyde; MSCs, mesenchymal stem cells.

As shown in Fig 5, AH group had prominently higher hepatic MDA (P<0.001; Fig 5A; S4A Table) but lower GSH (P<0.001; Fig 5B; S4B Table) levels than the control group. After i.p. transplantation of MSCs, the hepatic MDA level was markedly reduced (P<0.001; Fig 5A; S4A Table), and the hepatic GSH (P<0.001; Fig 5B; S4B Table) was significantly enhanced.

### MSCs decreased hepatic neutrophil and macrophage infiltration

**Fig 6:**
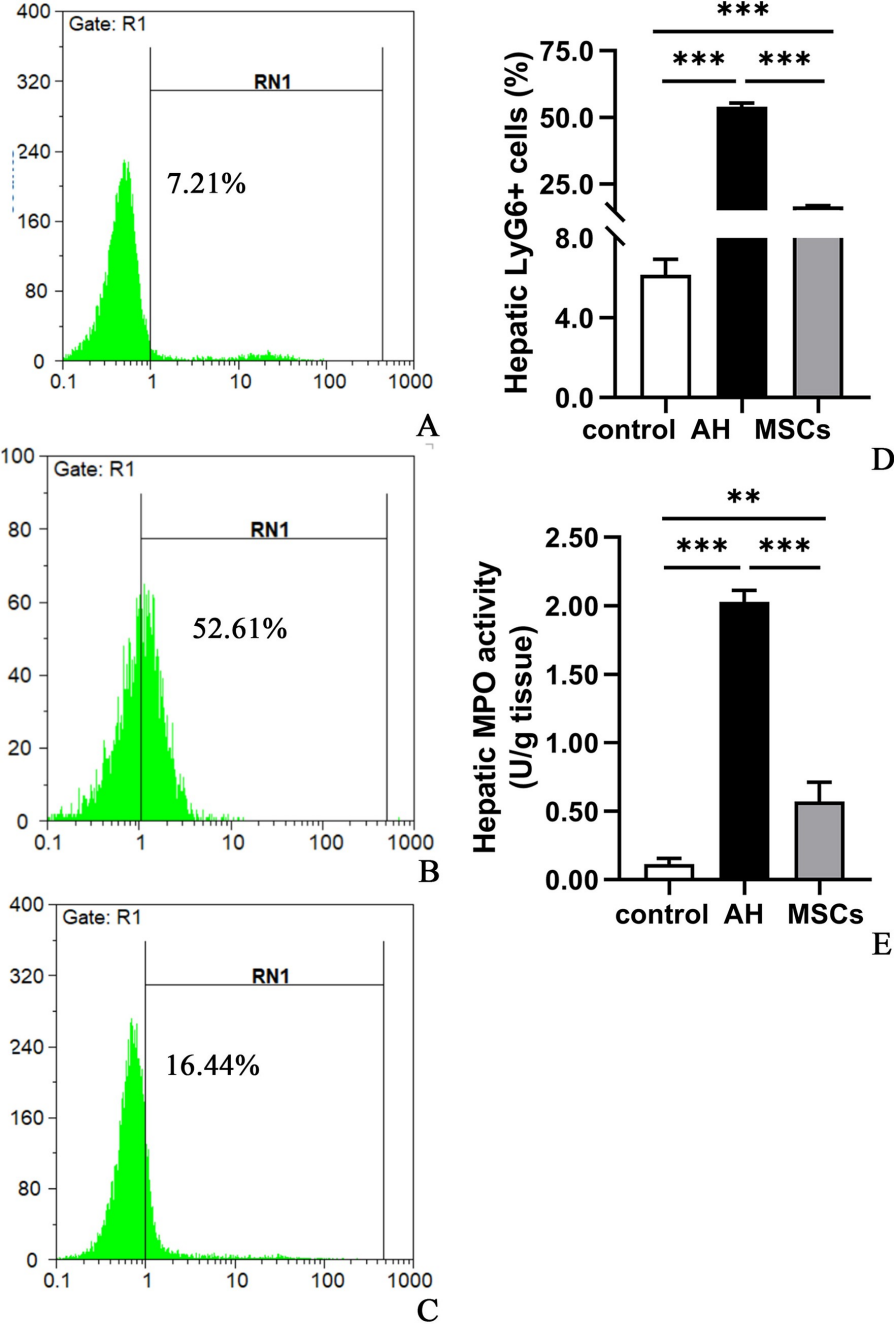
A-E MSCs decreased the percentage of neutrophils (Ly6G+) from the total isolated liver cells determined by flow cytometry and hepatic MPO level assay: control (A), AH (B) and MSCs groups (C); statistical analysis (D); hepatic MPO level (E). Four mice per group were used. *P < 0.05, **P < 0.01, ***P < 0.001. AH, alcoholic hepatitis; MPO, myeloperoxidase; MSCs, mesenchymal stem cells.

**Fig 7:**
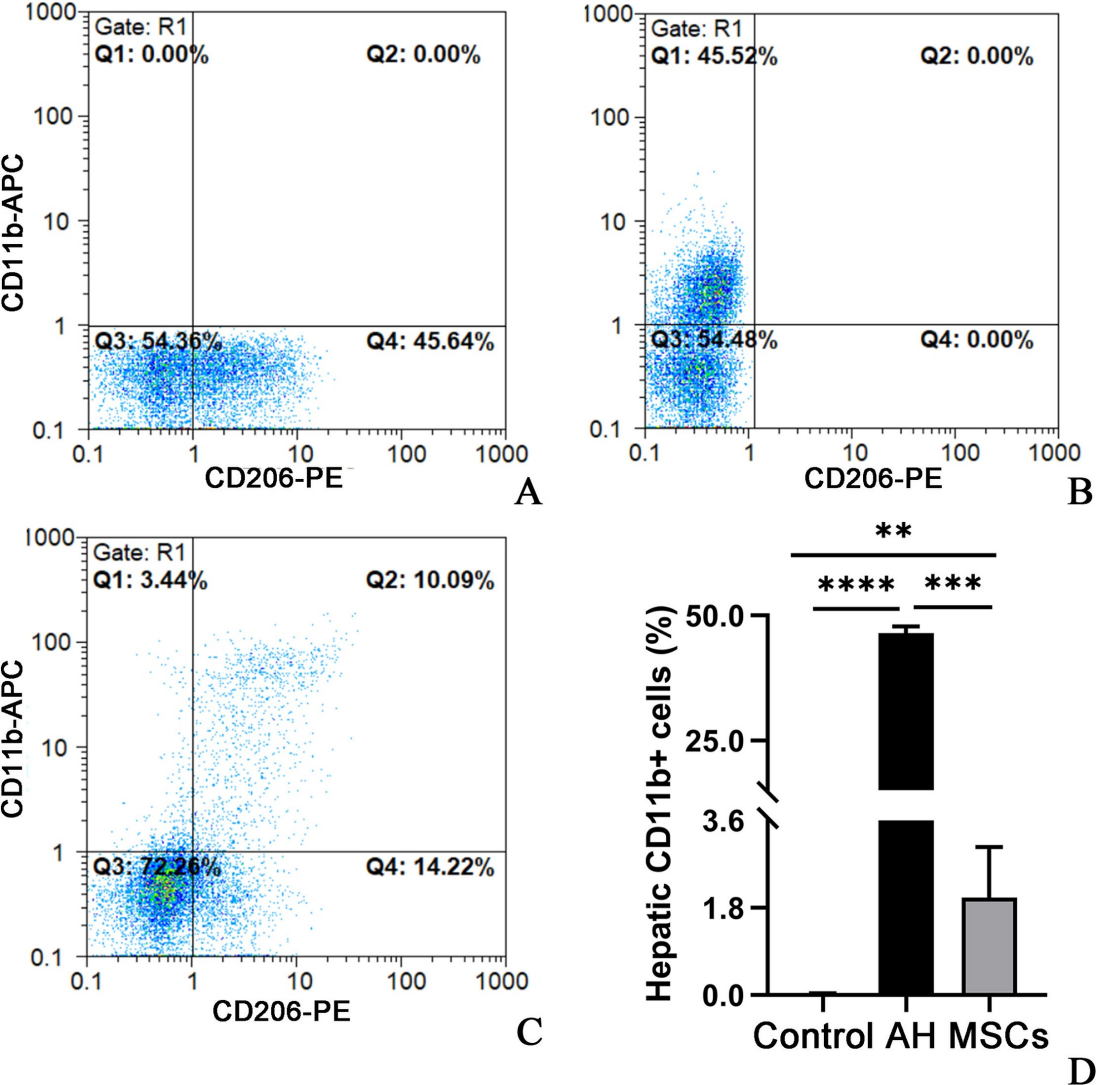
A-D MSCs decreased the percentage of macrophages (CD11b+) from the total isolated liver cells determined by flow cytometry: control (A), AH (B) and MSCs groups (C); statistical analysis (D). Four mice per group were used. *P < 0.05, **P < 0.01, ***P < 0.001. AH, alcoholic hepatitis; APC, allophycocyanin; MSCs, mesenchymal stem cells; PE, phycoerythrin.

Flow cytometric analysis demonstrated that neutrophil (Fig 6A–6D; S5A Table) and macrophage (Fig 7A–7D; S6 Table) infiltration was significantly greater in the AH group than the control group (P<0.001), which were markedly decreased after cytotherapy with MSCs (Figs 6 and 7; P<0.001). In agreement with the flow cytometric analysis, the hepatic MPO activity (Fig 6E; S5B Table), another marker of hepatic neutrophil infiltration [15,17], was also markedly higher in the AH group than the control group (P<0.001; Fig 6E; S5B Table), which were prominently reduced after MSCs treatment (P<0.001; Fig 6E; S5B Table).

## Discussion

In the present study, four important findings were presented here. First, we showed that i.p. transplantation of MSCs was effective in attenuating liver injury in AH mice. Second, we found that MSCs were able to reduce liver steatosis and hepatic lipid contents. Third, we demonstrated that MSCs could alleviate hepatic oxidative stress. Fourth, we observed that MSCs markedly decreased hepatic neutrophil and macrophage infiltration.

Previous studies showed that female rodents developed more florid liver damage than males after exposure to ethanol [22], and male mice were notoriously resistant to ethanol-induced liver injury [15], so we used female mice as the experimental animals to obtain more prominent liver injury. The NIAAA model for ALD has now been increasingly accepted [15–17], so we used this model to investigate the efficacy of MSCs. Serum ALT and AST levels are sensitive parameters reflecting liver injury. Previous studies showed that chronic alcohol feeding for 10 days in addition to a single binge dose of alcohol gavage (i.e. acute-on-chronic alcohol feeding) synergistically enhanced liver/body weight ratio, serum levels of ALT and AST [15–17]. In line with these studies [15–17], mice in the AH group had significantly higher liver/body weight ratio, ALT and AST levels than the control mice (Fig 2B–2D), with ALT and AST reaching a maximum of 279IU/L and 515IU/L, respectively. After i.p. transplantation of MSCs, thee liver/body weight ratio, ALT and AST levels were significantly decreased compared to mice with i.p. injection of sterile saline (Fig 2B–2D), which showed that i.p. transplantation of MSCs was effective in attenuating liver injury in AH mice.

In the past, a fatty liver was considered to be a benign condition, but now it’s a known risk factor for steatohepatitis, fibrosis, and hepatocellular carcinoma, because excessive lipid accumulation in liver may lead to liver injury due to direct cellular toxicity caused by lipid peroxidation, free fatty acids (FFAs), mitochondrial damage and oxidative stress [23,24]. Previous studies reported that acute-on-chronic alcohol feeding synergistically increased liver steatosis and hepatic fat deposition [15–17]. In agreement with these studies, our study found that mice in the AH group had more evident liver steatosis and elevated hepatic TC and TG levels than the control mice (Fig 3A–3F; Fig 4A and 4B), which were markedly reduced by i.p. infusion of MSCs. Reduce liver steatosis and hepatic fat contents may in turn contribute to improvement of liver injury [23,24], though the mechanism of how MSCs exert their antisteatosis effect remain unclear in our study. Interestingly, a recent study reported that i.p. modified human adipose tissue-derived MSCs with antioxidant genes Sod2 (mitochondrial) and catalase (cytosolic) upregulated using adenoviral constructs significantly reduced liver steatosis and TG content [25]. Presumably, MSCs may exert their antisteatosis effect through antioxidant effect and reduction of oxidative stress in our study.

Oxidative stress is known to play a critical role in the pathogenesis of ALD [26], which can induce steatosis, hepatomegaly, inflammatory cell infiltration, fibrosis and cirrhosis [27]. In our study, we observed marked hepatic MAD accumulation but GSH depletion in AH mice compared to control mice, which was consistent with previous studies [15,17]. After i.p. injection of MSCs, the hepatic MDA content was reduced and GSH level was enhanced significantly, compared to mice in the AH group (Fig 5A and 5B), which was in concert with a previous study that reported the antioxidant effect of MSCs derived from human umbilical cord in a mouse model of acetaminophen induced acute liver failure[28].

Neutrophil infiltration is a known hallmark of HA, which may exacerbate liver injury in AH mice [15,17]. To investigate the effect of MSCs on hepatic neutrophil infiltration, we measured the hepatic Ly6G+ cells using flow cytometry and hepatic MPO levels using a MPO spectrophotometric assay kit, as both Ly6G and MPO were known markers of neutrophils [15,17]. In conformity to previous studies [15,17], we found that AH mice had prominent neutrophil infiltration in the liver with Ly6G+ cells reaching reaching about 55% of the isolated liver cells, when compared to control mice (Fig 6A–6D). In line with the flow cytometric result, the hepatic MPO activity was markedly highly in the AH group than in the control (Fig 6E). However, i.p. transplantation of MSCs significantly decreased both hepatic Ly6G+ cells and MPO activity in the MSCs group, as compared to the AH group. These findings suggested that MSCs were able to suppress hepatic neutrophil infiltration. Notably, neutrophil infiltration may cause hepatocellular damage by killing hepatocytes through generation of oxidative stress and cytotoxic mediators [29,30].

We further measured the hepatic infiltration of macrophages, because previous studies reported that damage to hepatocytes could cause release of danger associated molecular patterns (DAMPs) that could activate the hepatic resident Kupffer cells [31], leading to production of inflammatory mediators and recruitment of immune cells including neutrophils and macrophages to the liver [32]. Our results showed that mice with AH had much greater hepatic macrophage infiltration (CD11b+ cells) than the control mice (Fig 7D), which was consistent with previous studies [15,16]. Similarly, i.p. transplantation of MSCs also significantly reduced hepatic macrophage infiltration (Fig 7D). All the above findings suggest that MSCs may alleviate liver injury via suppression of oxidative stress, hepatic infiltration of neutrophils and macrophages in AH mice.

## Conclusion

**Fig 8:**
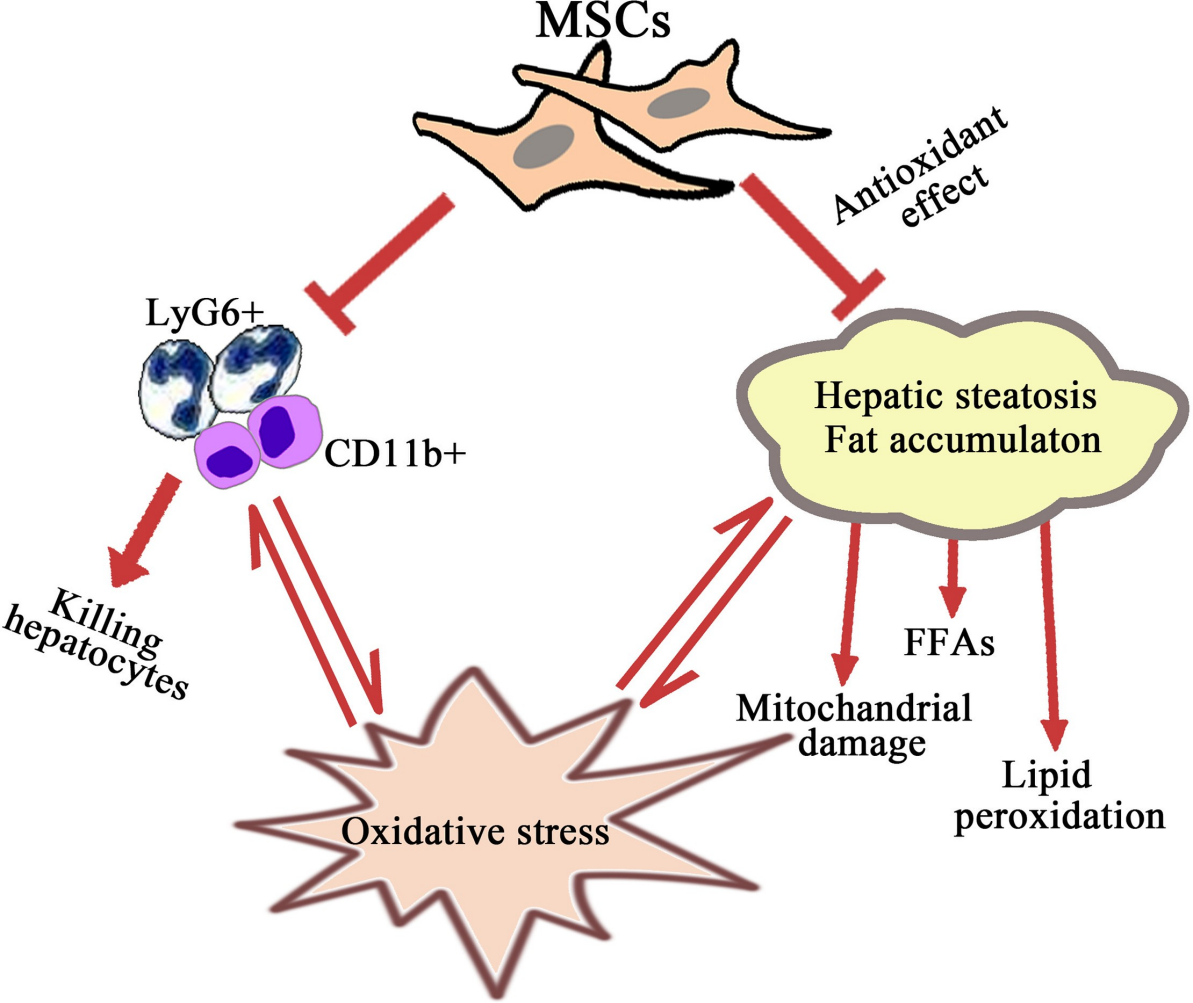
Schematic mechanism of mouse BM-MSCs activities in the treatment of alcoholic hepatitis. BM-MSCs, bone marrow-mesenchymal stem cells.

In summary, our study clearly showed that MSCs derived from mouse BM were effective in treating AH, and they might exert their hepatic protective effects via inhibiting neutrophil and macrophage infiltration and antioxidant effect. Based on the previous findings [23–25,27,29,30], we come up with the schematic mechanism underlying the mouse BM-MSCs activities in the treatment of AH (Fig 8). These findings may help in exploration of effective cytotherapies for the treatment of ALD.

## Supporting information

S1 Table Histological scoring system for alcoholic fatty liver disease. (DOCX)

S2 Table Body weight, liver to body weight ratio, serum ALT and AST. (DOCX)

S3 Table Hepatic lipid contents and histology scores. (DOCX)

S4 Table Hepatic MDA and GSH levels. (DOCX)

S5 Table Hepatic Ly6G+ cells and MPO levels. (DOCX)

S6 Table Hepatic CD11b+ cells. (DOCX)
