# Supplementary material for: Mesenchymal stem cells reduce alcoholic hepatitis in mice via suppression of hepatic neutrophil and macrophage infiltration, and of oxidative stress
Source: PLoS One. 2020 Feb 11;15(2):e0228889. doi: 10.1371/journal.pone.0228889 (PMC7012433; doi:10.1371/journal.pone.0228889)
Supplement: S1 Table — (DOCX) [file pone.0228889.s001.docx]

Table 1 Histological scoring system for alcoholic fatty liver disease

|  | Pathological feature  in a 100× field | Activity scores (0-3) |
| --- | --- | --- |
| Hepatocyte ballooning | None | 0 |
|  | Few ballooned cells | 1 |
|  | Many cells/prominent ballooning | 2 |
| Hepatic steatosis | <5% hepatocytes involved | 0 |
|  | 5–33% hepatocytes involved | 1 |
|  | 33–66% hepatocytes involved | 2 |
|  | >66% hepatocytes involved | 3 |
| Necroinflammatory activity | None | 0 |
|  | <2 foci per 100× field | 1 |
|  | 2-4 foci per 100x field | 2 |
|  | 5-10 foci per 100× field | 3 |
|  | >10 foci per 100× field | 4 |
