# Supplementary material for: Mesenchymal stem cells reduce alcoholic hepatitis in mice via suppression of hepatic neutrophil and macrophage infiltration, and of oxidative stress
Source: PLoS One. 2020 Feb 11;15(2):e0228889. doi: 10.1371/journal.pone.0228889 (PMC7012433; doi:10.1371/journal.pone.0228889)
Supplement: S2 Table — (DOCX) [file pone.0228889.s002.docx]

|  | Day 1 | Day 3 | Day 6 | Day 9 | Day 11 |
| --- | --- | --- | --- | --- | --- |
| Control | 17.8±1.2  (n=10) | 18.1±1.4  (n=10) | 19.0±0.9  (n=10) | 18.7±1.0  (n=9) | 18.5±0.8  (n=9) |
| AH | 17.9±1.2  (n=10) | 18.3±1.4  (n=9) | 18.5±1.5  (n=8) | 18.6±1.4  (n=7) | 18.7±1.1  (n=7) |
| MSCs | 17.8±1.1  (n=10) | 17.9±0.9  (n=9) | 18.5±0.9  (n=9) | 18.7±1.3  (n=8) | 18.7±0.8  (n=8) |
| *P*-value  (ANOVA) | NS | NS | NS | NS | NS |

A. Body weights (g) of mice in three groups.

B. Liver/body weights ratio of mice in three groups.

|  | Control (n=6) | AH (n=6) | MSCs (n=6) |
| --- | --- | --- | --- |
| 1 | 0.0564 | 0.0723 | 0.0564 |
| 2 | 0.0603 | 0.0685 | 0.065 |
| 3 | 0.0682 | 0.0726 | 0.0601 |
| 4 | 0.0625 | 0.0707 | 0.0624 |
| 5 | 0.0613 | 0.0634 | 0.0645 |
| 6 | 0.0543 | 0.0743 | 0.0568 |
| Mean | 0.06050 | 0.07030 | 0.06087 |
| Standard deviation | 0.004883 | 0.003906 | 0.003734 |

C. Serum ALT (IU/L) levels of mice in three groups.

|  | Control (n=5) | AH (n=5) | MSCs (n=5) |
| --- | --- | --- | --- |
| 1 | 49.3 | 279.2 | 99.3 |
| 2 | 54.8 | 221.3 | 90.7 |
| 3 | 55.3 | 213.9 | 98.7 |
| 4 | 57.1 | 205.9 | 110.4 |
| 5 | 50.1 | 268.3 | 101.2 |
| Mean | 53.34 | 237.7 | 100.1 |
| Standard deviation | 3.440 | 33.54 | 7.026 |

1. Serum AST (IU/L) levels of mice in three groups.

|  | Control (n=5) | AH (n=5) | MSCs (n=5) |
| --- | --- | --- | --- |
| 1 | 115.0 | 515.6 | 229.9 |
| 2 | 125.1 | 482.5 | 208.8 |
| 3 | 119.3 | 444.2 | 196.8 |
| 4 | 127.6 | 428.7 | 228.5 |
| 5 | 110.2 | 512.3 | 220.2 |
| Mean | 119.4 | 476.6 | 216.8 |
| Standard deviation | 7.196 | 39.21 | 14.00 |
