# Supplementary material for: Mesenchymal stem cells reduce alcoholic hepatitis in mice via suppression of hepatic neutrophil and macrophage infiltration, and of oxidative stress
Source: PLoS One. 2020 Feb 11;15(2):e0228889. doi: 10.1371/journal.pone.0228889 (PMC7012433; doi:10.1371/journal.pone.0228889)
Supplement: S3 Table — (DOCX) [file pone.0228889.s003.docx]

A. Hepatic TC content (mmol/g protein) of mice in three groups.

|  | Control (n=5) | AH (n=5) | MSCs (n=5) |
| --- | --- | --- | --- |
| 1 | 0.1203 | 0.9039 | 0.3611 |
| 2 | 0.1128 | 1.0535 | 0.2729 |
| 3 | 0.1277 | 0.8314 | 0.2583 |
| 4 | 0.119 | 0.8575 | 0.2945 |
| 5 | 0.1123 | 0.9123 | 0.3521 |
| Mean | 0.1184 | 0.9117 | 0.3078 |
| Standard deviation | 0.006305 | 0.08596 | 0.04647 |

µ

B. Hepatic TG content (mmol/g protein) of mice in three groups.

|  | Control (n=5) | AH (n=5) | MSCs (n=5) |
| --- | --- | --- | --- |
| 1 | 0.1477 | 1.3735 | 0.3393 |
| 2 | 0.1402 | 1.1432 | 0.4767 |
| 3 | 0.1303 | 1.108 | 0.321 |
| 4 | 0.1483 | 1.0768 | 0.3333 |
| 5 | 0.1321 | 1.4123 | 0.3412 |
| Mean | 0.1397 | 1.223 | 0.3623 |
| Standard deviation | 0.008454 | 0.1576 | 0.06445 |

C. Hepatocyte ballooning score of mice in three groups.

|  | Control (n=5) | AH (n=5) | MSCs (n=5) |
| --- | --- | --- | --- |
| 1 | 1.555555556 | 1.333333333 | 0.666666667 |
| 2 | 1.333333333 | 1.333333333 | 0.555555556 |
| 3 | 1.555555556 | 1.444444444 | 0.444444444 |
| 4 | 1.333333333 | 1.555555556 | 0.555555556 |
| 5 | 1.444444444 | 1.444444444 | 0.666666667 |
| Mean | 1.444 | 1.422 | 0.5778 |
| Standard deviation | 0.1111 | 0.09296 | 0.09296 |

1. Hepatic steatosis score of mice in three groups.

|  | Control (n=5) | AH (n=5) | MSCs (n=5) |
| --- | --- | --- | --- |
| 1 | 0.111111111 | 1.555555556 | 0.333333333 |
| 2 | 0.222222222 | 1.666666667 | 0.222222222 |
| 3 | 0.111111111 | 1.555555556 | 0.333333333 |
| 4 | 0.222222222 | 1.666666667 | 0.222222222 |
| 5 | 0.333333333 | 1.777777778 | 0.444444444 |
| Mean | 0.2000 | 1.644 | 0.3111 |
| Standard deviation | 0.09296 | 0.09296 | 0.09296 |

1. Hepatic necroinflammation score of mice in three groups.

|  | Control (n=5) | AH (n=5) | MSCs (n=5) |
| --- | --- | --- | --- |
| 1 | 0.222222222 | 1.444444444 | 0.333333333 |
| 2 | 0.333333333 | 1.555555556 | 0.222222222 |
| 3 | 0.222222222 | 1.444444444 | 0.333333333 |
| 4 | 0.333333333 | 1.333333333 | 0.444444444 |
| 5 | 0.111111111 | 1.555555556 | 0.333333333 |
| Mean | 0.2444 | 1.467 | 0.3333 |
| Standard deviation | 0.09296 | 0.09296 | 0.07857 |
