# Supplementary material for: Mesenchymal stem cells reduce alcoholic hepatitis in mice via suppression of hepatic neutrophil and macrophage infiltration, and of oxidative stress
Source: PLoS One. 2020 Feb 11;15(2):e0228889. doi: 10.1371/journal.pone.0228889 (PMC7012433; doi:10.1371/journal.pone.0228889)
Supplement: S4 Table — (DOCX) [file pone.0228889.s004.docx]

A. Hepatic MDA content (nmol/mg protein) of mice in three groups.

|  | Control (n=5) | AH (n=5) | MSCs (n=5) |
| --- | --- | --- | --- |
| 1 | 1.0059 | 8.6691 | 2.1316 |
| 2 | 0.907 | 10.1769 | 2.0722 |
| 3 | 1.0152 | 8.2003 | 3.2009 |
| 4 | 0.8847 | 8.5546 | 2.3912 |
| 5 | 1.0043 | 8.7241 | 2.0142 |
| Mean | 0.9634 | 8.865 | 2.362 |
| Standard deviation | 0.06227 | 0.7612 | 0.4906 |

µ

B. Hepatic GSH content (µmol/g protein) of mice in three groups.

|  | Control (n=5) | AH (n=5) | MSCs (n=5) |
| --- | --- | --- | --- |
| 1 | 108.8555 | 12.4049 | 64.1041 |
| 2 | 100.369 | 11.5262 | 71.8644 |
| 3 | 115.4646 | 10.2479 | 69.5785 |
| 4 | 111.2198 | 10.5433 | 77.3031 |
| 5 | 109.2121 | 12.1253 | 64.5924 |
| Mean | 109.0 | 11.37 | 69.49 |
| Standard deviation | 5.506 | 0.9487 | 5.471 |
