# Supplementary material for: Mesenchymal stem cells reduce alcoholic hepatitis in mice via suppression of hepatic neutrophil and macrophage infiltration, and of oxidative stress
Source: PLoS One. 2020 Feb 11;15(2):e0228889. doi: 10.1371/journal.pone.0228889 (PMC7012433; doi:10.1371/journal.pone.0228889)
Supplement: S5 Table — (DOCX) [file pone.0228889.s005.docx]

A. Percentage of hepatic Ly6G+ cells of mice in three groups.

|  | Control (n=4) | AH (n=4) | MSCs (n=4) |
| --- | --- | --- | --- |
| 1 | 5.43 | 52.61 | 16.44 |
| 2 | 7.21 | 55.56 | 16.84 |
| 3 | 6.27 | 54.78 | 16.36 |
| 4 | 5.67 | 53.21 | 16.48 |
| Mean | 6.145 | 54.04 | 16.53 |
| Standard deviation | 0.7930 | 1.365 | 0.2126 |

µ

B. Hepatic MPO (U/g tissue) of mice in three groups.

|  | Control (n=4) | AH (n=4) | MSCs (n=4) |
| --- | --- | --- | --- |
| 1 | 0.0947 | 1.9177 | 0.4044 |
| 2 | 0.1584 | 2.1027 | 0.5088 |
| 3 | 0.1363 | 2.0053 | 0.715 |
| 4 | 0.069 | 2.0867 | 0.6531 |
| Mean | 0.1146 | 2.028 | 0.5703 |
| Standard deviation | 0.04027 | 0.08506 | 0.1404 |
