# Supplementary material for: Mesenchymal stem cells reduce alcoholic hepatitis in mice via suppression of hepatic neutrophil and macrophage infiltration, and of oxidative stress
Source: PLoS One. 2020 Feb 11;15(2):e0228889. doi: 10.1371/journal.pone.0228889 (PMC7012433; doi:10.1371/journal.pone.0228889)
Supplement: S6 Table — (DOCX) [file pone.0228889.s006.docx]

Percentage of hepatic CD11b+ cells of mice in three groups.

|  | Control (n=4) | AH (n=4) | MSCs (n=4) |
| --- | --- | --- | --- |
| 1 | 0.02 | 48.38 | 1.34 |
| 2 | 0.03 | 46.32 | 1.13 |
| 3 | 0.00 | 45.52 | 3.44 |
| 4 | 0.04 | 45.69 | 2.12 |
| Mean | 0.02250 | 46.48 | 2.008 |
| Standard deviation | 0.01708 | 1.314 | 1.046 |

µ
